# Representing vision and blindness

**DOI:** 10.1186/s13326-016-0058-0

**Published:** 2016-03-30

**Authors:** Patrick L. Ray, Alexander P. Cox, Mark Jensen, Travis Allen, William Duncan, Alexander D. Diehl

**Affiliations:** Department of Philosophy, University at Buffalo, Buffalo, NY USA; New York State Center of Excellence in Bioinformatics and Life Sciences, University at Buffalo, Buffalo, NY USA; Department of Neurology, Jacobs School of Medicine and Biomedical Sciences, University at Buffalo, Buffalo, NY USA

## Abstract

**Background:**

There have been relatively few attempts to represent vision or blindness ontologically. This is unsurprising as the related phenomena of sight and blindness are difficult to represent ontologically for a variety of reasons. Blindness has escaped ontological capture at least in part because: blindness or the employment of the term ‘blindness’ seems to vary from context to context, blindness can present in a myriad of types and degrees, and there is no precedent for representing complex phenomena such as blindness.

**Methods:**

We explore current attempts to represent vision or blindness, and show how these attempts fail at representing subtypes of blindness (viz., color blindness, flash blindness, and inattentional blindness). We examine the results found through a review of current attempts and identify where they have failed.

**Results:**

By analyzing our test cases of different types of blindness along with the strengths and weaknesses of previous attempts, we have identified the general features of blindness and vision. We propose an ontological solution to represent vision and blindness, which capitalizes on resources afforded to one who utilizes the Basic Formal Ontology as an upper-level ontology.

**Conclusions:**

The solution we propose here involves specifying the trigger conditions of a disposition as well as the processes that realize that disposition. Once these are specified we can characterize vision as a function that is realized by certain (in this case) biological processes under a range of triggering conditions. When the range of conditions under which the processes can be realized are reduced beyond a certain threshold, we are able to say that blindness is present. We characterize vision as a function that is realized as a seeing process and blindness as a reduction in the conditions under which the sight function is realized. This solution is desirable because it leverages current features of a major upper-level ontology, accurately captures the phenomenon of blindness, and can be implemented in many domain-specific ontologies.

## Background

The human visual system is a complex, organized collection of specialized cells and structures that function together to encode and represent one type of stimulus (light). Light enters the system through the eye, a complex organ that includes structural, optical, and muscular components, which operate together to ensure an intact focused image reaches the retina. In the retina, two types of photoreceptor cells encode visual information in the form of light by converting it to a neuronal signal. A network of synapses connect the photoreceptor cells via bipolar neurons, ganglion cells, horizontal cells, and amacrine cells to the optic nerve, which connects to the brain proper. Many of the fibers of the optic tract innervate at the lateral geniculate nucleus in the posterior thalamus while others project to the superior colliculus. The lateral geniculate nucleus sends projection cell axons to the visual cortex. The superior colliculus, in addition to signals from the retina, receives indirect signals from the visual cortex. The visual cortex is composed of layers that are responsible for different tasks. Most of the input from the lateral geniculate nucleus occurs in layer IV of the primary visual cortex. Cells in layer IV are connected in patterns that work to integrate visual data from the lateral geniculate nucleus into a cognitive representation of objects in the visual field [1].

Because the human visual system involves many anatomical structures and complex functions, it can fail in a multitude of ways. Such failures can occur in any of the various structures that make up the system, and often result in visual impairment or blindness. Blindness has been studied from multiple perspectives, whether as the result of accident or disease. Most of the associated anatomical entities are well described, and many of the affected processes involved in visual perception are well understood; yet the representation of the fundamental nature of blindness from an ontological perspective remains incomplete.

In its most basic form, blindness is the impairment of visual function below a certain threshold. Where this threshold is set varies from context to context, and standards for blindness vary across international and institutional borders. The World Health Organization characterizes blindness as visual acuity of less than 20/500 or a visual field of less than 10 degrees. In the United Kingdom, the Certificate of Visual Impairment characterizes blindness as visual acuity of less than 20/400. In the United States, the American Medical Association characterizes blindness as visual acuity of less than 20/200 or a visual field of less than 20 degrees. There have also been recent calls by the International Council of Ophthalmology to define blindness and visual impairment according to their own standards, at least part of which involve visual substitution skills employed by persons [2]. This expansion to include visual substitution skills in the account of blindness is important because it demonstrates that visual acuity only represents one dimension of blindness. Other types of visual impairments fall outside the scope of visual acuity—such as the ability or inability to differentiate colors.

The relationships between blindness, visual impairment, and visual acuity can be difficult to describe without resort to fiat definitions, such as those described above, which offer little insight into the underlying nature of the phenomena. We start with the premise that blindness is a specific type of visual impairment and that blindness results from a failure in the visual process. Visual acuity is the specific ability an individual has with respect to vision (how well or poorly an individual is able to visually process stimuli—or perhaps the sharpness of the representations of external stimuli by an individual). Many of the problems associated with representing blindness result from a conflation of closely associated concepts about the vision process and its absence. By identifying the entities in reality, the components of the visual system and their associated functions and processes, we may therefore sort out what blindness is, and what blindness results from.

Current efforts to define terms related to blindness, while systematic, are rather problematic. The National Health Interview Service (NHIS) offers a definition of *'an individual who suffers from vision loss'* as “those who report they have trouble seeing [3].” The problem with such a definition is that self-assessment is a reliably poor criterion for sensory perception. People are often mistaken about their abilities and skills regarding their perception. An older NHIS survey, the National Health Interview Survey on Disability (NHIS-D) implemented a sharp cut-off for those who are legally blind, a visual acuity of 20/200 or less. The American Community Survey (ACS) provides a definition of those with *'difficulty seeing'* as individuals who self-report either blindness or “serious difficulty seeing even when wearing eyeglasses [3].” Along with the aforementioned problem with self-assessment we now have an additional problem of qualification with corrective lenses. (Notice that this definition is too vague in that even a person with exceptional vision could have serious difficulty seeing when wearing glasses with a strong prescription; we surely would not want to label such a person as having difficulty seeing).

Governmental organizations implement their own standards for blindness. The Department of Labor's Bureau of Labor Statistics employs a Current Population Survey as a means for identifying those who have serious vision loss or blindness. The standard, once again, is one of self-assessment. “People with vision loss were identified by the CPS if they reported that they or someone in their household is blind or has serious difficulty seeing when wearing eyeglasses [3].” One of the problems with such wide-ranging definitions or criteria of blindness is that there seems to be no one standard. As a result it is very difficult to determine precisely how many people suffer from blindness or vision loss.

From a diagnostic perspective, the National Eye Institute (NEI) defines ‘blindness’ as “the best-corrected visual acuity of 6/60 or worse (=20/200) in the better seeing eye [4].” The American Optometric Association follows the protocol of the World Health Organization in defining ‘total blindness’ as “no light perception” and ‘near total blindness’ as “a best-corrected visual acuity of less than 20/1000 [5].” The standard United States legal definition of ‘blindness’ follows the criterion endorsed by the NEI, as a best-corrected visual acuity of 20/200 or worse.

The problems that arise for a definition of ‘blindness’ date to at least the beginning of the 20th century. N. Bishop Harman writes of a proposed definition of ‘blindness’, “By no possible phrasing can we present a sufficiently simple definition that will embrace the possible variations in these several factors that make up sight, and state that such and such a variation shall be accounted blindness [6].” Other, more recent assessments of the feasibility of defining ‘blindness’ have been met with similar reluctance but somewhat more optimism. “The fact is, however, that the briefest analysis of any dictionary definition will reveal the word ‘blind’ to be neither straightforward nor respectable [7]”.

The aforementioned problems notwithstanding, there are still difficulties surrounding blindness as a subject of investigation. If we understand blindness as the impairment of visual functioning beyond a certain threshold and accept that there are many different ways in which visual functioning can be impaired, then there are many different ways in which one may be blind. Furthermore, there are many different mechanisms that will cause blindness. Moreover, if we focus on the different types of visual impairment with respect to the features of the world that are not effectively represented in the visual process, ‘blindness’ can be used to describe these different types of failures of representation along the lines of features of the world (e.g. color blindness). This complicates our task because it shows that not only are there many different ways one can become blind, but different ways in which one may be blind. The nature of blindness is more complicated than it appears at first glance. Not only are there complications with respect to contextualized thresholds for determining blindness, but also difficulty in examining the natures of the differing types of visual impairment that lead to blindness.

There are two obvious and immediate challenges for representing and defining visual impairment and blindness. First, different groups use different standards of measurement. Second, different standards of classification can be used in conjunction with a single standard of measurement. These challenges make it difficult to share, reuse, and compare data on blindness and vision related disorders. In addition, there are more complex problems that arise in representing blindness in formal ontology. This paper explores the difficulties that arise in representing blindness ontologically and proposes a novel solution to representing visual impairment and blindness in a formal manner. While we believe that the definition of ‘blindness’ (and its related terms) will not come easily, our goal in this paper is to provide an ontological representation of ‘blindness’ through a careful examination of the nature of blindness and related phenomena.

### Current ontological representations of vision and blindness

The Gene Ontology defines ‘visual perception’ as “The series of events required for an organism to receive a visual stimulus, convert it to a molecular signal, and recognize and characterize the signal. Visual stimuli are detected in the form of photons and are processed to form an image [8].” Thus, visual perception, or seeing, is a relational process between an agent and the stimulus itself. The process of seeing is representational insofar as the agent represents the stimulus in some manner (we leave the source of this stimulus and the nature of this representation to further examination). We may characterize the diminishment or cessation of this relational process as blindness or a loss of vision.

Currently there are relatively few ontologies that contain the term ‘blindness’ and fewer still that offer a well-formed definition of ‘blindness’. There are no realism-based ontologies that represent the phenomena surrounding blindness in a manner that reflects its complexity. Results of previous attempts to characterize blindness using current ontologies are listed in Table 1. Of the attempts to represent blindness in biomedical ontologies, it is a popular strategy to classify blindness as a phenotype [9].

**Table 1:** Previous attempts to define and represent blindness

*Ontology*	*Term*	*Definition*	*Parent Class*
Gene Ontology (GO)	Visual perception	The series of events required for an organism to receive a visual stimulus, convert it to a molecular signal, and recognize and characterize the signal. Visual stimuli are detected in the form of photons and are processed to form an image.	Sensory perception of light stimulus
GO	Detection of visible light	The series of events in which a visible light stimulus is received by a cell and converted into a molecular signal. A visible light stimulus is electromagnetic radiation that can be perceived visually by an organism; for organisms lacking a visual system, this can be defined as light with a wavelength within the range 380 to 780 nm.	Detection of light stimulus
GO	Detection of light stimulus involved in visual perception	The series of events involved in visual perception in which a light stimulus is received and converted into a molecular signal.	Visual perception
GO	Determination of sensory modality	The determination of the type or quality of a sensation. Sensory modalities include touch, thermal sensation, visual sensation, auditory sensation and pain.	Sensory processing
Mammalian Phenotype (MP)	Blindness	Loss of the sense of sight.	Abnormal vision
MP	Abnormal vision	Inability or decreased ability to see.	Abnormal eye physiology
MP	Decreased visual acuity	Loss of visual acuity or ability to distinguish small details	Abnormal visual acuity
Human Disease Ontology (DOID)	Blindness	N/A	Retinal disease
DOID	Color blindness	A blindness that is characterized by the inability or decreased ability to see color, or perceive color differences, under normal lighting conditions.	Blindness
Human Phenotype (HP)	Blindness	Blindness is the condition of lacking visual perception due to physiological or neurological factors.	Visual Impairment

Biomedical ontologies that seek to represent phenotypes typically rely on Entity-Quality (EQ) methodology [10]. The EQ methodology leverages the existing structure of ontologies to generate a schema where the subject of the phenotype (the entity) is described by the phenotype that inheres in that entity (the quality). This approach is advantageous in that it provides a computational resource for researchers working with phenotypic qualities. This allows researchers to leverage reasoners where they were not leveraged before. In addition, the EQ method allows that “[phenotypes be] recorded using multiple ontologies in a highly expressive and finely detailed manner while maintaining correct logic and computability [10].” This is a very powerful and innovative method used within the biomedical ontology community.

The EQ methodology works well for phenotypes and qualities that inhere in entities. For example, if we wanted to say, “some *human being has red eyes”*, we could accomplish this via EQ methodology by leveraging terms from the Uber Anatomy Ontology (UBERON) and the Phenotype and Trait Ontology (PATO). We would use the terms ‘red’ from PATO and ‘eye’ from UBERON and apply the EQ methodology to yield EQ = UBERON:eye + PATO:red [10].

While this solution has its advantages, its shortcomings outweigh its advantages for the subject of blindness. The main problem with applying the EQ method to blindness is that it is unclear whether blindness is a phenotype. The Ontology for General Medical Science (OGMS) provides the following definition for ‘phenotype’:

A (combination of) quality(ies) of an organism determined by the interaction of its genetic make-up and environment that differentiates specific instances of a species from other instances of the same species [11].

If blindness is a phenotype and phenotypes are qualities (or combinations thereof), then blindness is a quality (or combination thereof). If blindness is a quality (or combination thereof), then blindness is a specifically dependent continuant that needs no further process in order to be realized. But this consequence is incorrect—blindness is the inability of the vision function to be realized. Blindness is not a quality of a visual system or of an organism that the visual system is part of. Blindness is a failure in the realization of the visual function.

Another option to consider is an alternative definition of ‘phenotype’. One standard definition for ‘phenotype’ is that a phenotype is the outcome of a given genotype in a particular environment [12]. If it is the case that blindness is a phenotype and that a phenotype is the outcome of a given genotype in a particular environment, then blindness is the outcome of a given genotype in a particular environment. Of course this poses no problems when we are discussing instances of blindness with a genetic basis. However, some instances of blindness do not have a genetic basis, but rather are the result of acute trauma or some other non-genetic phenomenon. Since there are cases of blindness that have non-genetic bases, it is clear that blindness is not a phenotype in all cases according to this definition of ‘phenotype’. Since blindness is not a phenotype in all cases (if any), we should look for an alternative account of blindness.

One of the general problems with the treatment of blindness as a phenotype (quality) is that it is inconsistent with the proposed definitions provided in these accounts. If blindness is a lack of sight (or vision) and sight (or vision) is a realizable entity, then blindness should be the lack of a process or the lack of some realizable entity. If blindness is a phenotype and therefore a type of quality, then there is a quality that is a lack of a realizable entity. But this is impossible. There cannot be an entity that is both a lack of a realizable entity and a quality at the same time. The lesson here is not that blindness does not exist but rather that blindness is something other than a phenotype.

If blindness is not a phenotype, what other ontological solutions are there? One of the most likely candidate solutions involves using the Human Disease Ontology (DO). The Human Disease Ontology currently does not provide a definition of 'blindness', but we could propose a plausible candidate on their behalf following their characterization of color blindness as an inability or decreased ability to detect light stimulus. 'Color blindness' is defined in DO as: “a blindness that is characterized by the inability or decreased ability to see color, or perceive color differences, under normal lighting conditions [13].” (Table 1) Moving from this definition of a specific type of blindness to a general definition of blindness should produce the result that blindness is “the inability or decreased ability to see or perceive, under normal lighting conditions”.

While this is a generally attractive view, it does not stand up to careful examination. In the first place, DO categorizes blindness as a disease. Blindness is not a disease though it may result from a particular disease. Moreover, it is not a type of retinal disease as DO currently represents it. There are many diseases that may result in blindness and many diseases can complicate the sightedness of individuals, but blindness is not itself a disease. Furthermore, blindness need not result from a disease. It may instead be caused by a single event, as is the case with acute trauma or flash blindness. Blindness also need not be limited to problems in the retina. Cortical blindness is a type of blindness that does not involve any malfunction with the retina. As detailed in the last section, certain types of blindness are not limited to just one mechanism of realization, or to realization in only one location.

These problems notwithstanding, the more pressing concern with this solution is that there does not seem to be any indication of what an inability or decreased ability would be. If abilities are dispositions or functions, then they are realizable entities. The concern is plain—according to BFO a realizable entity cannot lack. Realizable entities cannot present in degrees, as their existence is an all-or-nothing affair. If blindness is an inability to detect light, then all cases of blindness will be a complete inability to detect light stimulus, which fails to capture the cases of blindness that are not the complete inability to detect light stimulus. If blindness is a decreased ability to detect light, then it cannot be represented as a decreased function or disposition in BFO. But, if sight is a function and blindness is the lack of sight, then an account of blindness as an inability cannot be given either. Hence, we believe that this type of account is confused.

Moving away from an account based on the DO representation, another route for capturing blindness is to maintain that blindness is a disorder, where 'disorder' is defined by OGMS as “[a] material entity that is clinically abnormal and part of an extended organism [11].” The problem with this approach is that it is unclear that blindness is a material entity. If one thinks that blindness is the absence of the sight function, then it does not seem that blindness is a material entity (absences of functions are not material entities). Further, one cannot point to a material entity and identify it as blindness because blindness is not spatially extended and spatial extension is a hallmark of material entities. For these reasons, blindness cannot be a disorder per OGMS.

### The realist approach to blindness

There are several lessons to be learned from this concise review of the representations of blindness in biomedical ontologies. First, it is rather difficult to characterize an entity via a lack or absence, which seems to be the case with blindness (the lack of sight) [14]. Consider the paradigm case of an ontological absence involving material entities: a hole. There does not seem to be anything to which one can attribute characteristics. In the Basic Formal Ontology (BFO), holes are continuant entities susceptible to characteristic ascription like material entities, but are essentially non-material. This indicates at least a *prima facie* problem with characterizing entities via a lack. BFO is an upper-level ontology that is realist, fallibilist, perspectivalist, and adequatist [15]. The core of BFO lies in its distinction between continuants and occurrents, which tracks the difference between the two different types of fundamental entities in the world. BFO is currently used as the upper-level ontology for many biomedical ontologies and is the backbone of the Open Biological and Biomedical Ontologies collaborative [16].

A second problem arises when we consider attempts to capture the existence conditions of a hole. If a hole is defined by a lack (where the lack is both necessary and sufficient), then there are all sorts of things that would qualify as a hole. For example, if one were to characterize a hole as a lack of matter surrounded by matter, then the interior of a room would count as a hole, as would the inside of a bag. A strategy has been implemented in anatomy for representing lacking a part; however, it is contentious whether it will translate well for things that are not material entities, such as processes or functions 1 [17]. The reason that such a strategy will not work well for non-material entities is that the *lacks_part* relation does not apply to functions because functions do not have parts. 2 Processes, on the other hand, do have parts but the parts are temporal parts, not material entities (processes can include material entities as participants, but these are not themselves parts of processes).

Blindness does not seem to yield a precise definition or even clearly differentiated conditions under which it is present or absent, apart from the fiat standards discussed previously. Blindness often presents gradually which is commonly due to degeneration of the eye or apparatuses associated with vision. As a result, many cases of blindness are progressive and it is exceedingly difficult to determine at which point blindness has come into existence. In addition to these complications, there is controversy over the threshold for blindness. Hence, it is common for publications regarding blindness to specify which definition of ‘blindness’ they employ [18].

Even given these complications regarding blindness, we contend that it is useful to give a univocal account of the phenomenon for purposes of ontological development. Such an account should capture all or a vast majority of the cases of blindness and the various classifications of blindness found in the literature. Thus, the account should remain general and flexible enough to capture a wide range of characterizations yet it should also be rigid enough to remain informative and insightful.

There are many types of blindness. For example, there is color blindness and change blindness. Blindness can also be defined relative to a context. An individual might be legally blind but still be able to detect some light stimulus. Similarly, one might be visually impaired to the extent that they are prohibited from flying a jet aircraft but not from driving. Hence, we might say that someone is ‘blind according to [x]’ where [x] is some standard of evaluation for sightedness. In this sense, blindness comes in degrees. The extent to which someone has a lack of vision will be graded. If we think of seeing as a relational process between an agent who is representing and the thing represented and assess the accuracy of such representations on a scale of 1 to 0 (complete representational veracity to no representational veracity), the ability of the person to see will be somewhere on the continuum from 0 to 1—the closer to 0 one’s representation of the stimulus is, the more blind that individual is.

Lending to the confusion surrounding the status of blindness (and vision) is the method used for assessing visual acuity. Typically, visual acuity is expressed as a relationship between two values—the distance a subject stands from an optical chart and the distance at which a normal subject would stand from the chart to discern the same visual detail. Putting aside the problems associated with this particular type of visual acuity assessment, we have discussed above how this can lead to confusion regarding what conditions are indicative of blindness [18].

Given the above considerations, one might conclude that there is not a single coherent ontological category that corresponds to what blindness is as an entity. Instead, blindness could be an amalgam of loosely related entities or something that is not ontologically well-formed. We find this conclusion unsatisfactory. It is useful for clinicians and researchers to have a coherent theory of blindness that encompasses the range of conditions commonly understood to be forms of blindness. We simultaneously realize that blindness seems to be characterized as relative or context-sensitive (the term itself might be context-sensitive or the phenomenon might be context-sensitive or both). We favor the view that the term ‘blindness’ denotes a single phenomenon reflecting severe visual impairment relative to a particular context of evaluation. Thus, ‘blindness’ denotes an ontologically well-formed category.

According to BFO, a function is a type of disposition. A disposition is a realizable entity whose realization occurs in virtue of the bearer’s physical constitution and because the bearer is in some special physical circumstances. A function is a disposition that exists as the product of natural selection or intentional design on the part of the agent. Because functions are dispositions they are realizable entities. They are realized as processes that are sometimes called ‘functionings’. Functions are regarded as non-accidental in BFO meaning that they come into being because of natural selection or intentional design [19]. All of the functions a given entity possesses are intimately tied to the type of entity under examination, whether the entity is biological or artifactual. Functions are internally-grounded realizable entities. An internally-grounded realizable entity “…is a realizable entity that is a reflection of the (in-built or acquired) physical make-up of the *material entity* in which it inheres [19].” Changing the physical structure of an entity that bears a given function might alter the realization of the function in question.

A reason to believe that sight is a disposition is that it is realized by processes grounded in a material entity. This is a hallmark of a disposition (of which functions are a special type) as described above. We have another reason to think that sight is a disposition: the fact that if sight ceases to exist, then the bearer is physically changed. Although the entities still have the disposition to see, they are blind because they can no longer realize that disposition due to some change in their physical constitution.

Furthermore, we believe that sight is a function of a visual system (or at least of visual systems of entities with higher-order cognitive functions). One reason is that development of the ability to see is the result of an evolutionary process. The physical makeup of particular organisms have changed and adapted over time to select for the existence of the sight function. For non-biological entities possessing sight, if any, the sight that they possess is not accidental, but rather a product of intentional design on the part of the creator. For these reasons, we contend that sight is a function.

It is important to note here that it is not the case that for all instances of structural change in the bearer of a function that the function ceases to be realizable. In other words, it is not the case that every physical change in a bearer alters the function in question. It is also the case that some bearers of functions can undergo changes that render them unable to realize a function while the function persists in its bearer. For example, one of the functions of the human heart is to pump blood. Physical changes to the heart may cause the heart to be unable to perform its function—a large tear in a ventricle wall might be such a physical change. Other physical changes might not cause the loss of the ability to perform a function—a slightly enlarged section of muscle might be such a physical change. But in all cases where a function ceases to exist or comes into being there must be a corresponding physical change. To use an artifactual example, a radiator has the function to disperse heat. Certain physical changes to a radiator will render the radiator unable to realize its function, such as the presence of corrosion on the cooling fins or a cracked regulator valve. But in this case we would not say that the radiator has lost its function, rather we would say that its function cannot be realized. If there were a sufficiently significant physical modification of the right kind to an entity, only then would we contend that the function ceases to exist. This is an important point that will play a significant role later in our discussion of blindness.

We can characterize the specific type of function (sight) by identifying and describing its defining features. Employing such a strategy, we provide an account of sight as the function to receive photons and interpret them as visual information. Similarly, we can characterize seeing as the process by which photons contacting the retina are coded into an action potential and interpreted as visual information. Having given an account of vision as the realization of the sight function, it is then natural to identify the processes by which the sight function is realized as vision. There are many other functions involved in the realization of the sight function as described above. We are omitting detailed discussion of these functions for ease of exposition.

If we are correct in our claim that sight is a function, and that blindness is related to the realization of this function, then it seems that we can develop our understanding of the phenomena of blindness through the characterization of sight as a function. Because blindness is a wide-ranging phenomenon (i.e., there are many types of blindness), it would behoove us to explore the range of blindness types in an attempt to fully capture blindness.

### Color blindness

Color perception is possible for humans because we (typically) have three different types of cone cell photoreceptors that detect photons within corresponding ranges of wavelength. Photons within the range of a particular type of cone cell photoreceptor cause an action potential in the corresponding cone cell and the action potential is sent along eventually to the primary visual cortex. Color is coded by the differentiation of the firing of these types of cone photoreceptor cells.

Color blindness is a condition wherein an individual has an inability to distinguish between two or more colors. In some cases photons of differing color spectrum wavelength are represented or interpreted as the same when they are distinct. In other cases, an individual cannot report a difference between two or more wavelengths of photons [20]. The inability to distinguish between two or more types of light is not limited to just one cone type [21]. Complicating this picture somewhat is that there are many mechanisms identified as causes of color blindness and that these mechanisms are not localized to one anatomical region. Some color blindness is due to an individual lacking cone cells or a certain type of cone cell. Other times the cause is cortical [22]. For example, the complete lack of the ability to distinguish colors, achromatopsia, can have its underlying causal mechanism located within the retina (congenital achromatopsia) or the visual cortex (cerebral achromatopsia). Thus color blindness is similar to other types of blindness in that its causes and the mechanisms associated with it are diverse and complex.

### Flash blindness

Flash blindness is a type of blindness that results from sudden exposure to bright light—such as the bright flash that results from the flash bulbs on older cameras or the detonation of an atomic weapon. The sudden influx of light oversaturates the photopigments of the retina and the individual becomes unable to convert photons to a neural signal [23]. Flash blindness is commonly temporary, where the subject regains their full ability to see within a few minutes. There are some extreme cases, however, where flash blindness results in permanent vision loss [24].

### Inattentional blindness

Inattentional blindness can be described by an individual’s lack of perception of a salient object or feature in their visual field due to lack of attention [25]. Investigation into the mechanism for this phenomenon has revealed both a nonconscious and conscious component. The nonconscious mechanism includes contour integration in the early visual cortex while conscious integration includes involvement from the lateral occipital cortex and is mediated more strongly by focused attention [26].

### Proposed solution

Drawing on the lessons from the previous sections, we propose a solution to the problem that blindness poses for ontology development. Because sight is a function and blindness is seemingly the non-realization of that function, we set forth an account of blindness where blindness is a reduction of the trigger conditions under which the sight function is realized. To understand this, we need to understand how dispositions are related to triggering conditions. “A disposition is a causal property that is linked to a realization, i.e., to a specific behavior which the individual that bears the disposition will show under certain circumstances or as a response to a certain stimulus (trigger) [27]”.

This solution is able to deal with the cases outlined above. Color blindness is a reduction in the (color) trigger conditions under which a vision function is realized. Although different types of color blindness will involve different types of reduction of conditions, they are unified as a single phenomenon by the fact that they all involve the reduction of the number of particular light wavelengths that result in differentiated visual representation. For flash blindness, we say that there is a temporary (or possibly permanent) reduction in the conditions under which the vision function is realized, whatever the mechanism realizing the function of sightedness may be. It may be that cases of temporary flash blindness are impairments of functions in the following manner: they are grounded in a physical change that results in a malfunction but not a (permanent) loss of a function. Because the function is realized by a rather complicated functioning in both cases, the type of blindness can range over different types of failure in functioning so long as the reduction of conditions is similar.

The case of inattentional blindness is interesting and problematic. It is unclear whether the inattentional blindness is a type of blindness or the use of the term ‘blindness’ is metaphorical. If it is not the case that inattentional blindness is a type of blindness, then we need not worry about its formalization. If, however, intattentional blindness is a type of blindness, then we propose the following formalization: inattentional blindness is an instance of a non-realized disposition (the vision function is non-realized). The disposition will not be realized in one of two ways: nonconsciously by some mechanism of contour integration in the early visual cortex or consciously by failure of function by the lateral occipital cortex. This is why inattentional blindness is much like more traditional types of blindness even though its mechanism and presentation differs from these traditional types of blindness. One could also classify types of blindness by the types of failure in sight functionings, if one so chose.

Having provided an informal account of what constitutes blindness we now provide a formalization of the solution. Because our account relies heavily on triggers, dispositions, and processes realizing those dispositions, any formalization will have to invoke all of these at a minimum. We can characterize triggers, dispositions, and processes realizing dispositions in the following manner. Dispositions exist outside of their realizations—the disposition of fragility inheres in a vase whether or not it there is a process of shattering to realize that disposition. Some dispositions exist without any realizations—that is to say, there are some dispositions that will never be realized because the triggering conditions are not met. Realizations are processual entities and have as participants the material entities. Dispositions and qualities inhere in material entities. For dispositions, then, we can identify the following features.

### Dispositions, triggers, and background conditions

The relationship between triggering conditions, dispositions, and realizations described above is over-simplified. Keep in mind that the dispositional account given characterizes dispositions as non-probabilistic and their associated phenomena as straightforwardly accessible. In reality, many dispositions are most likely probabilistic and the precise nature of their associated phenomena (triggering conditions, realization processes) is currently unknown. This should not deter one from seeking to give an account of dispositions, triggers, and realization processes, however. One of the more attractive accounts proffered is from Rohl and Jansen [27]. According to this account, there is a primitive (undefined) relation (has_trigger_R_) that holds between the triggering process and the realization process. The relationship between the disposition and the triggering process is defined in terms of that primitive relationship as follows:^3^

$$ \mathrm{d}\kern0.5em \mathbf{has}\_\mathbf{trigge}{\mathbf{r}}_{\mathbf{D}}\mathrm{t}\ \mathrm{i}\mathrm{f}\mathrm{f}:\ \mathrm{t}\mathrm{here}\ \mathrm{exists}\ \mathrm{some}\ \mathrm{r}\ \left(\mathrm{d}\mathbf{has}\_\mathbf{realization}\kern0.5em \mathrm{r}\ \mathrm{and}\ \mathrm{r}\kern0.5em \mathbf{has}\_\mathbf{trigge}{\mathbf{r}}_{\mathbf{R}}\mathrm{t}\right) $$

Roughly, this says that some particular disposition has a particular trigger just in case there is some realization process such that the particular disposition has that realization process and that realization process has some trigger. We would then say that the particular disposition in question has that particular trigger. This is an instance-level relationship. An example of such a relationship would be the fragility of a vase. The particular disposition a vase has for shattering is realized by some instance of a realization process (a shattering process), which is triggered by some particular triggering event, e.g., a dropping process. The relationship between the particular realization process that realizes the disposition and the process that triggers or precedes the realization process is irreducible.

Once there is a relationship between the instances of dispositions and triggers, it is rather straightforward to extend this to types of dispositions. Following Rohl and Jansen, this relationship can be captured in the following manner:

$$ Dhas\_ trigge{r}_DT{=}_{\mathrm{DEF}}\forall \times \left(\times \kern0.5em \mathbf{instance}\_\mathbf{of}D\kern0.5em \supset \kern0.5em \forall \mathrm{y}\ \left(\times \kern0.5em \mathbf{has}\_\mathbf{trigge}{\mathbf{r}}_{\mathbf{D}}\mathrm{y}\kern0.5em \supset \kern0.5em \mathrm{y}\kern0.5em \mathbf{instance}\_\mathbf{of}\kern0.5em T\right)\right) $$

This definition is at the level of types. It specifies the relationship between disposition types and triggering process types. In a similar manner the relationship between realization types and triggering types can be captured:

$$ R\  has\_ trigge{r}_RT{=}_{\mathrm{DEF}}\mathrm{f}\mathrm{o}\mathrm{r}\kern0.5em \forall \kern0.5em \times \left(\times \kern0.5em \mathbf{instance}\_\mathbf{of}R\kern0.5em \supset \kern0.5em \exists \mathrm{y}\left(\mathrm{y}\kern0.5em \mathbf{instance}\_\mathbf{of}T\& \times \mathbf{has}\_\mathbf{trigge}{\mathbf{r}}_{\mathbf{R}}\mathrm{y}\right)\right) $$

This relationship holds between realization process types and triggering process types. Since dispositions involve triggering processes, realization processes, and dispositions themselves, it is essential to capture the relationships between these three entities. The above relationship specifications do just that. To see how this works in our case of vision, consider the following relation for the vision of an individual:

Agent x’s disposition for vision has_trigger_D_ particular lighting conditions iff: there exists some neural processes of agent x (agent x’s disposition to see has_realization neural processes of agent x and neural processes of agent x has_trigger_R_ particular lighting conditions).

The relationships would then exist at the universal level:

*Vision Disposition has_trigger*_*D*_*Lighting Conditions (range of light wavelengths)* = _DEF_ ∀x (x instance_of *Vision Disposition* ⊃ ∀y (x has_trigger_D_ particular lighting conditions ⊃ particular lighting conditions instance_of *Lighting Conditions (range of light wavelengths*)).

*Photon to Neural Signal Transduction has_trigger*_*R*_*Lighting Conditions (range of light wavelengths)* = _DEF_ for ∀x (x instance_of *Photon to Neural Signal Transduction* ⊃ ∃y (y instance_of *Lighting Conditions (range of light wavelengths)* & x has_trigger_R_ y)).^4^

The above specifications may be complex but have a number of advantages. First, they capture the processes that underlie dispositions and the dispositions themselves, which allows for more accurate modeling. Second, this approach accounts for all of the processes (or at least all of the relevant processes) involved in dispositions. Approaches that do not account for all of the relevant processes involved in dispositions will be incomplete. Third, it leverages BFO as an upper-level ontology and inherits all of the benefits therein. Namely, using BFO provides a realist framework for modeling biomedical entities of interest and provides pre-theoretical interoperability with other biomedical ontologies that also employ BFO as an upper-level ontology.

### Possible objections

One possible objection to this account is that since blindness is a reduction in the range of conditions under which a disposition is realized, blindness is extrinsic to the agent that is blind. So, according to our account, turning off the lights is sufficient to make someone blind. The thrust of this objection being that the reduction of triggering conditions is extrinsic to the agent, but blindness is a phenomenon that is intrinsic to an agent. Therefore our account is incorrect

We think that this objection is confused. The confusion here is one between the presence of the conditions and the range of conditions. To take an example, water has a disposition to freeze at or under a certain temperature at a given level of atmospheric pressure (which just happens to be a range of conditions under which the freezing process is realized). The temperature constitutes a triggering condition for the freezing disposition. Lowering the temperature brings the disposition closer to realization due to the change in the external environmental conditions, but it does not mean that water freezes at a lower temperature. Nor does this change the disposition that water has to freeze. It is not that the range of conditions that changed, rather that the conditions themselves changed. The conditions under which a disposition is realized are external to an entity, much like the range, but these two things are independent of one another. Conditions themselves can change without the range of conditions changing, as is the case with reductions in triggering conditions. In this way we can see that turning off the lights does not constitute a reduction in the range of conditions for which a sight function is realized. Rather, it only constitutes a reduction in the actual lighting conditions in that situation.

There is an important point to be gleaned from this objection, however, in regard to a concern over the distinction between intrinsic and extrinsic properties and whether blindness is intrinsic or extrinsic. We have no reason to suppose that blindness is either intrinsic or extrinsic, at least not pre-theoretically. The reduction in the range of conditions under which the vision function is realized is due to a change in the physical basis of the bearer of the function. This, we believe, accounts for the intuition that blindness is an intrinsic property. Indeed our account of blindness is compatible with this observation, so those who believe that blindness is intrinsic should not be hostile to our view. We leave further discussion of intrinsic/extrinsic for a later and more detailed paper.

A second objection runs along the following lines: the solution offered fails to adequately distinguish a failure of seeing (or sight) from the inability to see. In the case of inattentional blindness one is failing to see something but yet they have the ability to see that thing. This is an important distinction: in fact it is the very difference between being blind and being able to see perfectly well. It is only when one lacks the ability to detect visual stimuli that they are blind. The account provided confounds these two phenomena and is thus incorrect.

We think this objection raises a very important point that we have alluded to earlier. We agree that there is a distinction to be made between having the ability to see and failing to notice a feature of the world versus not having the ability to see. The former typically would not be considered a case of blindness but the latter surely would. As we have stated before, the case of inattentional blindness is interesting and problematic. We have covered the possibility that inattentional blindness is a case of blindness and given an account of how that should be formalized. If the envisioned case driving the objection is one where the use of ‘blindness’ is metaphorical, then we need not and should not explain inattentional blindness as a type of blindness. If, on the other hand, inattentional blindness is not a metaphorical blindness (it is an instance of a non-realized visual disposition), then it will be formalized in the manner other types of blindness are formalized according to this schema. That is, the disposition will not be realized in one of two ways: nonconsciously by some mechanism of contour integration in the early visual cortex or consciously by a failure of function by the lateral occipital cortex.

In the case where one fails to see not through an inability to realize a disposition, but rather a simple mistake of perception (the objection at hand), this would firmly fall into a case of metaphorical blindness. If it is the case that all instances of inattentional blindness are such failures to see not because of an inability to realize a disposition, but rather by a mistake of perception, then inattentional blindness is a metaphorical blindness. If not, then we have a formalization at the ready.

A third objection can be described in the following way: One way to explain blindness is that there are many sub-processes that participate in the more complex process of vision. When one of these sub-processes does not occur, then the vision process does not occur. What is happening in the vision case is complicated, at least in part because there are fine points regarding processes, dispositions (functions), and their respective sub-processes and sub-dispositions (sub-functions). It is the case that many of the large, easily observable dispositions seem to be not single dispositions but rather many dispositions working together. It is more accurate to say that the realization of many dispositions is a complex of processes, some of which may themselves constitute realizations of dispositions that, in a way, seem to be sub-dispositions. Indeed when we consider complex systems like the human visual system it seems that this more complex picture is closer to the truth than the simple explanation proffered by the canonical dispositional picture. For example, the human visual system contains many components and those components each have distinct functions—the function of the retina is to convert photons to action potentials and the function of the optic nerve is to transmit the action potential to the lateral geniculate nucleus and the function of the primary visual cortex is to integrate the action potentials and organize that information in such a way that it is intelligible for a human being, etc. Each of these processes could fail to be realized due to a failure of some mechanism, and those are the causally responsible entities in the case of blindness or impaired vision. When we think of these smaller processes and the material entities they are dependent upon, we see that color blindness is a temporary or permanent loss of one of the dispositions of one or more structures of the visual system by their physical alteration. This view is simpler because we can explain the disposition of an organism by appealing to their parts and the realization of sub-processes, which is more accurate than simply appealing to larger-scale dispositions.

We find the approach described in the previous paragraph problematic for a few reasons. First, the nature of dispositions in BFO is such that the account this response posits is insufficiently robust. As we have discussed, dispositions are such that they cannot be within the domain or range of the *lacks_part* relationship. If there is a loss of a disposition or function, we have to determine how to represent a loss of a disposition, which is not something we can do simply by leveraging existing relationships.

Second, one of the problems for such an account is the nature of functions, which are a special type of disposition in BFO. It is the case that functions can be lost under certain circumstances, i.e., under circumstances of extreme physical, structural change of the bearer of the function. However, it is not the case that any physical change in a bearer of a function such that the function ceases will entail the loss of the function. This is the case in BFO as well as cases of vision under examination in this paper.

For example, a door has a function to open. It is essential to the proper functioning of the door that its function is only realized under certain conditions. If the door opens too easily (under a range of conditions that is too wide, say such that it opens under the pressure of the wind), we would still contend that the door bears the function to open, but that it is malfunctioning (due to a faulty latch or some such). Likewise, a door can be difficult to open while retaining its function to open, also due to some structural alteration. But we would still say that the door has the function to open (and close) and that it is malfunctioning. For each of these examples the door retains its function but the function is realized under a smaller (or larger) range of conditions (i.e., it takes more or less force to open the door). The range of conditions under which a function is realized is reduced because of an alteration in physical structure of the bearer of the function. The function of the entity is not altered, however; its functioning is. In this way a difficult door is similar to some cases of blindness, in that both of the entities bearing the function retain that function even though the function is realized under a broader or narrower range of conditions.

Now, it could be appropriate to frame such an event in terms of the various sub-processes or processes realizing the function (what are called functionings) not occurring, and this is indeed what is happening. But such a framing would fail to accurately capture all of a single type of phenomenon, in our case blindness. Further, what unites all cases of blindness is not that some functioning or another is not occurring, but that we have as a result a reduction of the range of conditions under which a disposition is realized. Any such reduction (under a certain threshold) is a case of blindness, no matter what sub-functioning is unrealized. Of course we may find further subtypes of blindness exist and these may be demarcated according to the various underlying physical mechanisms that reduce the range in conditions under which the vision function is realized, but this is consistent with our view. In fact it is our view. In each of the cases discussed, the difference is the mechanism underlying the reduction in the range of triggering conditions for the disposition of vision.

## Discussion

Given the problems associated with the currently existing accounts of blindness, we thus propose a new definition of blindness: blindness is a reduction of the trigger conditions under which the sight function is realized. If visual impairment admits of degrees, then it cannot be a function or disposition. Blindness cannot be a lack of a disposition or function because many things lack the function yet should not be classified as blind. The best option available for defining blindness is to say that sight is the function to receive photons and interpret them as visual information and then proceed to define blindness as a reduction of the conditions under which the disposition is realized.

One of the consequences of this reasoning is that many of the terms in various phenotype ontologies mistakenly refer to entities that are dispositions rather than qualities. Our goal in this paper is not to demonstrate that many terms in phenotype ontologies are mistakenly characterized as denoting qualities rather than dispositions, but merely that blindness is not a phenotype. We think that, as is consistent with BFO, dispositions are not phenotypes and phenotypes are not dispositions. It would be a mistake to confuse phenotypes and dispositions. This does not entail that there are no terms in various phenotype ontologies that denote dispositions, only that representing a disposition as a phenotype is incorrect from the perspective of BFO. While there may have been efforts to annotate data using *'blindness'* in phenotype ontologies in the past, we feel these efforts are mistaken.

Our approach has certain advantages. First, it accounts for the graded nature of blindness. The slow and sometimes gradual onset of blindness raises special problems for ontology construction as it admits of degrees and seemingly vague boundaries. Second, it classifies sight as an internally-grounded realizable entity, which makes use of the framework provided by an upper-level ontology such as BFO. Third, it is ontologically innocent in that there are no new entities to countenance in any upper-level ontology. The entities referenced by our solution are already present in BFO so there is no need to introduce new entities.

## Conclusions

The motivation of this project is to provide a simple yet flexible ontological account of blindness. Since blindness can result from a variety of diseases, the construction of ontologies that incorporate both blindness and the diseases that cause blindness, either directly or indirectly, is of importance to the biomedical community. But this is not a purely classificatory exercise—the employment of conditions under which a disposition (or function) is realized is a novel application of a tool that has been available for ontological developers for some time. It is the opinion of these authors that this type of usage could yield further fruitful results.

## Footnotes

1 Functions in BFO are a special type of disposition.

2 Functions do not have parts but the entities that have functions do have parts.

3 Note that the relationship has_trigger is two-place due to the limitations of formal ontology languages like OWL, which is only able to handle two-place relationships.

4 We leave bound variables uninstantiated for the reader.
